# Vertical ground motion and its effects on liquefaction resistance of fully saturated sand deposits

**DOI:** 10.1098/rspa.2016.0434

**Published:** 2016-08

**Authors:** Vasiliki Tsaparli, Stavroula Kontoe, David M. G. Taborda, David M. Potts

**Affiliations:** Department of Civil Engineering, Imperial College, London, UK

**Keywords:** vertical motion, liquefaction, bidirectional loading, variable permeability, post-liquefaction settlements

## Abstract

Soil liquefaction has been extensively investigated over the years with the aim to understand its fundamental mechanism and successfully remediate it. Despite the multi-directional nature of earthquakes, the vertical seismic component is largely neglected, as it is traditionally considered to be of much lower amplitude than the components in the horizontal plane. The 2010–2011 Canterbury earthquake sequence in New Zealand is a prime example that vertical accelerations can be of significant magnitude, with peak amplitudes well exceeding their horizontal counterparts. As research on this topic is very limited, there is an emerging need for a more thorough investigation of the vertical motion and its effect on soil liquefaction. As such, throughout this study, uni- and *bidirectional* finite-element analyses are carried out focusing on the influence of the input vertical motion on sand liquefaction. The effects of the frequency content of the input motion, of the depth of the deposit and of the hydraulic regime, using variable permeability, are investigated and exhaustively discussed. The results indicate that the usual assumption of linear elastic response when compressional waves propagate in a fully saturated sand deposit does not always hold true. Most importantly post-liquefaction settlements appear to be increased when the vertical component is included in the analysis.

## 1. Introduction

Shear waves and their vertical propagation through a level ground deposit have been a subject of extensive research over the years. This has improved the understanding of the physical mechanism and their effects on site amplification and sand liquefaction. On the other hand, vertical acceleration has drawn very limited attention with current design guidelines and site response analyses focusing only on the implications of the horizontal motion.

**Table 1.** Nomenclature.

*A*_o_	dilatancy constant
*a*_1_	defines the ratio of the elastic minimum over the elastic maximum shear modulus, *G*_min_/*G*_max_
*B*	small-strain stiffness shear modulus constant
*C_i_*	coefficients of the Fourier amplitude
(*e*_CS_)_ref_	critical state void ratio at a reference pressure $p_{\mathrm{ref}}^{\prime }$
*e*_max_	void ratio limit on the determination of the plastic modulus
*e*_o_	void ratio after consolidation
FA	Fourier amplitude
*f_i_*	frequencies in the discrete fast Fourier transform
*G*_s_	specific gravity of soil particles
*G*	shear modulus
*g*	gravitational acceleration
*H*_o_	fabric index constant
*h*_o_	plastic modulus constant
*K*	bulk modulus
*K*_o_	coefficient of earth pressure at rest
*k*	permeability
$k_{c}^{b}$	effect of *ψ* on the position of the bounding surface
$k_{c}^{d}$	effect of *ψ* on the position of the dilatancy surface
*k*_max_	maximum permeability at the time of liquefaction
*k*_o_	initial static permeability as measured in conventional laboratory testing
${\text{M}}_{c}^{c}$	critical state strength (ratio = $q/p^{\prime }$) in triaxial compression
${\text{M}}_{e}^{c}$	critical state strength (ratio = $q/p^{\prime }$) in triaxial extension
*m*	radius of the yield surface of the bounding surface plasticity model
*n*_k_	controls the effect of $r_{p}$ on the permeability
*p*	pore water pressure
$p_{o}^{\prime }$	mean effective stress level after consolidation
$p_{\mathrm{ref}}^{\prime }$	reference pressure (i.e. at atmospheric pressure)
$p_{\mathrm{YS}}^{\prime }$	determines the location of the secondary yield surface
q	triaxial deviatoric stress
$r_{p}$	mean effective stress ratio
$r_{p}^{*}$	cut-off mean effective stress ratio value at which the permeability attains its maximum value, *k*_max_
*r*_u_	excess pore water pressure ratio
SA	spectral acceleration
*S*_r_	degree of saturation
*T*_m_	mean period of ground motion
*u*	solid phase displacement
*α*	determines the effect of the elastic tangent shear modulus on the plastic modulus
*β*	determines the effect of the distance to the bounding surface on the plastic modulus
*γ*	determines the effect of void ratio on the plastic modulus
*γ*_sat_	saturated bulk unit weight
*γ*_1_	cut-off strain for the degradation of the elastic shear modulus
Δ*f*	frequency step in the discrete fast Fourier transform
$\Delta p^{\prime }$	change in mean effective stress
Δ*t*	time step
Δ*u*	change in pore water pressure
*ζ*	determines the effect of principal stress on fabric index
*κ*	parameter controlling the nonlinearity of the degradation of the elastic tangent shear modulus
*λ*	slope of critical state line in $e-\ln\;p^{\prime }$ space
*μ*	determines the effect of $p^{\prime }$ on the plastic modulus
*v*	Poisson's ratio
*ξ*	exponent for power law for critical state line
$\sigma_{h}^{\prime }$	horizontal normal effective stress
$\sigma_{v}^{\prime }$	vertical normal effective stress
$\sigma_{\mathrm{vo}}^{\prime }$	vertical normal effective stress after consolidation

However, an abundance of field observations on ground motions indicate that vertical acceleration can attain very high values at surface in the near field and can occasionally be accompanied by compressive structural damage [1–3]. Unexpectedly, high vertical ground accelerations have been recorded in past earthquake events, such as Northridge, California, 1994 and Kobe, Japan, 1995, in which liquefaction was also evident [2,4–6]. More recently, the 2010–2011 Canterbury earthquake sequence in New Zealand strongly corroborates the fact that there may be a relation between high vertical components of acceleration and soil liquefaction [7]. The effects of the 22 February 2011 seismic event in Christchurch were severe; extensive liquefaction and re-liquefaction of sandy deposits were observed, causing numerous casualties [8–11]. Compressive structural damage was also evident due to the high vertical accelerations registered, with peak surface amplitudes well exceeding a value of 1*g* [3,12].

Over the years there has been limited numerical research on the effects of vertical loading on soil liquefaction, with the conclusions drawn often indicating that no substantial effect exists: Ghaboussi & Dikmen [13] were among the first ones to carry out finite-element (FE) analyses to evaluate the seismic response and liquefaction potential of a horizontally layered soil deposit. From the analyses, the authors concluded that the resistance to liquefaction was not significantly affected by the vertical base acceleration. The latter resulted in some high-frequency oscillations in the evolution of pore water pressures, but no additional ones at the end of the strong motion. These will be subsequently termed residual pore water pressures. Shiomi & Yoshizawa [14] who carried out numerical analyses involving all three components of ground motion also came to similar conclusions.

Subsequently, Yang *et al*. [15] modelled an 18 m deep hypothetical level ground loose sand deposit to investigate numerically the effect of different levels of shaking on liquefaction resistance when the vertical motion is included in the simulation. The results showed that for all levels considered, the vertical motion was significantly amplified at ground level. Stiffness degradation was not evident in the fundamental frequency of the deposit in compression, while, again, only high-frequency oscillations were observed in the pore water pressure time histories due to the inclusion of the vertical motion.

Yang [16] further investigated the impact of the vertical ground motion on soil liquefaction by extending his analyses to model a partially saturated sand deposit below the ground water table level (GWTL) with a degree of saturation $S_{r}=99\%$, using an equivalent bulk stiffness for the pore fluid. Contrary to the full saturation case, the results showed that even a small reduction in the degree of saturation of the sand deposit, $S_{r}$, can substantially increase the rate of excess pore water pressure development and the amount of residual pore pressures when the vertical motion is included in the analyses. Nevertheless, even a small reduction in $S_{r}$ was shown to result in a significant decrease in the overall residual value of the pore pressure ratio (as a result of both the horizontal and vertical components) compared with the full saturation condition and hence, to a much higher resistance to liquefaction.

Stimulated by the above studies, this paper concerns a *numerical* investigation of the role of the vertical seismic motion on the physical mechanism of liquefaction. To this end, a hypothetical fully saturated level ground sand deposit is considered giving particular emphasis on the frequency content of the input excitation. Notably, two input motions with substantially different frequency ranges are used as the base excitation in nonlinear elasto-plastic fully coupled FE site response analyses. The first two parts of the study focus on the effects of frequency content and depth of the deposit, whereas in the third part variable permeability analyses are carried out to investigate drainage effects, the impact of viscous damping due to fluid–solid interaction and the influence of vertical motion on post-liquefaction settlements.

## 2. Modelled sand deposit and input ground motions

The numerical study models a hypothetical soil deposit consisting of Fraser River Sand (FRS) with a relative density of 40% and a permeability of 4.2 × 10^−4 ^m s^−1^. The value of permeability was obtained from laboratory tests conducted at the University of British Columbia on FRS [17]. The sand deposit was assumed to be fully saturated with the water table located at ground level, underlain by impermeable rigid bedrock. Two depths to bedrock were considered; a shallow deposit of 40 m depth and a deep one of 166 m depth.

The material properties for FRS are presented in table 2. The maximum elastic shear modulus follows a nonlinear distribution with depth according to the Hardin & Richart [18] expression. Based on the assumed variation of the maximum shear stiffness modulus, the average *small-strain* shear wave velocity is 166 m s^−1^ for the 40 m depth deposit and approximately 239 m s^−1^ for the 166 m deep deposit. The compressional wave velocity is mainly controlled by the bulk stiffness of the water (2.2 × 10^6^ kPa) and as such, is fairly similar in both deposits: 1611 m s^−1^ for the former and 1636 m s^−1^ for the latter. Given the above, the average non-degraded fundamental frequency of the 40 m deep FRS deposit is 1.06 Hz and 10.3 Hz for shear wave (S-wave) and compressional wave (P-wave) propagation, respectively. Similarly, the corresponding non-degraded fundamental frequencies for the 166 m deep deposit take average values of 0.36 and 2.46 Hz.

**Table 2.** Material properties for FRS.

properties	value
specific gravity of soil particles (*G*_s_)	2.720
initial void ratio (*e*_o_)	0.812
saturated bulk unit weight (*γ*_sat_)	19.120 kN m^−3^
earth pressure coefficient at rest (*K*_o_)	0.440
Poisson's ratio (*v*)	0.200
permeability (*k*)	4.200 × 10^−4 ^m s^−1^

In order to investigate the effect of the input motion on liquefaction occurrence, two ground motions of profoundly different frequency content were used in the study. The first one is the outcrop motion registered during the 22 February 2011 seismic event in Christchurch, New Zealand, characterized by a magnitude of *M*_w_ = 6.2. The motion was obtained from the Geonet database of geological hazards in New Zealand [19] and was recorded in the Lyttelton Port Company (LPCC) strong ground motion station (SMS), located 10 km southeast of the city of Christchurch. The area is believed to be underlain by a volcanic rock outcrop, although, as it lies in private land, the exact surface stratigraphy is unknown [20]. Despite these uncertainties, as this forms a theoretical study, the recorded LPCC motion was considered appropriate for the purposes of this investigation.

The second motion used was supplied by the Institute of Earth Science, Taiwan, and occurred on 20 May 1986, Lotung, Taiwan; a seismic event with an estimated local magnitude of *M*_L_ = 6.5 [21]. The motion was recorded at 47 m depth in a downhole array.

**Figure 1.**
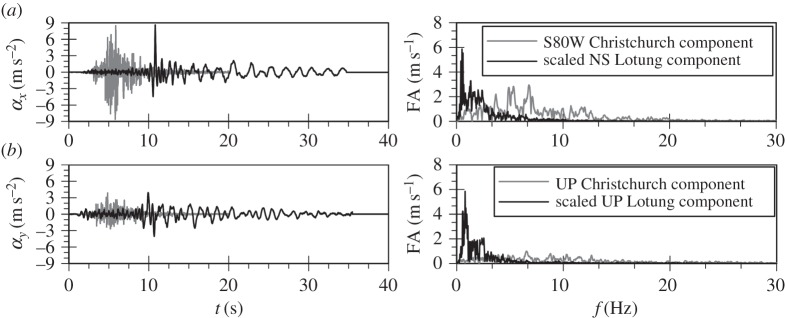
Acceleration time histories and Fourier spectra of input ground motions. (*a*) Horizontal components and (*b*) vertical components.

The acceleration time histories as well as the Fourier spectra of the horizontal and vertical components of the two ground motions are shown in figure 1. Baseline correction has been carried out for all four records using the computer software SeismoSignal v. 5.1.0 [22]. The Christchurch event is characterized by a peak horizontal (PHA) and a peak vertical (PVA) acceleration of 0.87*g* and 0.4*g*, respectively, with a ratio, PVA/PHA, of 0.45. The corresponding values for the Lotung event are 0.1*g* and 0.03*g*, resulting in a ratio of 0.33, however, in order to isolate the impact of the frequency content both components of the Lotung event were scaled up, so that its PHA and PVA matched those of the Christchurch event.

As shown in the Fourier spectra in figure 1, the amplitudes of the scaled up Lotung components are larger, but are distributed within a very narrow band of frequencies compared to the Christchurch components. The latter exhibit significant amplitudes up to 25 Hz. This difference can also be clearly seen from the mean periods, $T_{m}$, of the two events: 0.23 s for the Christchurch event compared with 1.07 s for the Lotung event. The value of $T_{m}$ is based on the following equation by Rathje *et al*. [23]:

2.1 $$T_{m}=\frac{\Sigma_{i}\cdot C_{i}^{2}\cdot (1/f_{i})}{\Sigma_{i}\cdot C_{i}^{2}},$$

for $0.25\;\mathrm{Hz}\leq f_{i}\leq 20\;\mathrm{Hz}$ and Δf≤0.05 Hz, where $C_{i}$ are the coefficients of the Fourier amplitude and $f_{i}$ and Δf are the frequencies and the frequency step, respectively, in the discrete fast Fourier transform.

## 3. Numerical procedure and constitutive model

Nonlinear elasto-plastic effective stress-based FE analyses were carried out with the u-p hydro-mechanically coupled formulation of the Imperial College Finite Element Program (ICFEP, [24]). The mesh represents a soil column, assuming plane strain conditions, consisting of either 160 × 1 (40 m deep deposit) or 664 × 1 (166 m deep deposit) eight-noded quadrilateral elements with pore water pressure degrees of freedom at the four corner nodes. The dimensions of the elements are 0.25 × 0.25 m^2^, with the height chosen such that, considering the frequency content of the two motions, it satisfies the recommendations by Bathe [25]. As stiffness degradation can be significant in liquefaction problems, an estimation of the degree of nonlinearity was obtained through preliminary equivalent linear analyses based on the ground motions and soil properties under consideration. As a result of such analyses, a 20% reduced stiffness compared with its small-strain value was used in element size calculations. In order to ensure one-dimensional soil response for level ground conditions, tied degrees of freedom are used at the lateral boundaries [26]. Additionally, for the horizontal or vertical motion dynamic analyses the displacements are restricted at the base of the mesh in the vertical or horizontal direction, respectively, while no restriction is imposed for *bidirectional* dynamic analyses. In terms of the hydraulic regime, pore water pressure degrees of freedom at the lateral boundaries and of the same elevation are tied to be equal, the flow is restricted at the base of the mesh, while zero pore water pressures are prescribed at the top nodes [24], therefore, allowing drainage only through the surface. The input motion is applied as an acceleration time history incrementally to the nodes located on the bottom boundary. A modified Newton–Raphson scheme employing a sub-stepping stress point algorithm forms the basis of the nonlinear solver [24], while the generalized α-method of Chung & Hulbert [27] is used as the time-integration scheme [28,29]. For accuracy purposes, a time step of Δ*t* = 0.01 s was found to be adequate for the Lotung components, whereas in the case of the Christchurch event, due to the wider frequency range, Δ*t* had to be reduced to a value of 0.003 s.

The mechanical behaviour of the sand is modelled using a two-surface bounding surface plasticity model. This is based on the Papadimitriou & Bouckovalas [30] modified version of the original two-surface model proposed by Manzari & Dafalias [31]. The model has been implemented in ICFEP in generalized three-dimensional stress space and includes a number of alterations targeted at improving various aspects of its capabilities [32,33]. These include a power law for the Critical State Line, an altered expression of the hardening modulus and the introduction of a secondary yield surface to improve the numerical stability of the model.

The model parameters for FRS are presented in table 3, as established by Klokidi [34]. A total of 63 drained and undrained monotonic triaxial compression and extension element tests, as well as 21 cyclic drained and undrained direct simple shear tests were available for the calibration procedure [35–37]. The meaning of the model parameters is explained in detail in Taborda [32] and Taborda *et al*. [33] and is not repeated herein for brevity.

**Table 3.** Model parameters for Fraser River Sand [34].

model	value	model	value	model	value
$p_{\mathrm{ref}}^{\prime }\;(\mathrm{kPa})$	100.00	*A*_o_	1.00	*h*_o_	0.119
(*e*_CS_)_ref_	0.95	*m*	0.065	*γ*	1.016
*λ*	0.05	$p_{\mathrm{YS}}^{\prime }\;\left(\mathrm{kPa}\right)$	1.00	*e*_max_	0.97
*ξ*	0.60	*B*	422.00	*α*	1.00
${\text{M}}_{c}^{c}$	1.38	*a*_1_	0.44	*β*	0.00
${\text{M}}_{e}^{c}$	1.00	*κ*	2.00	*μ*	1.00
$k_{c}^{b}$	2.50	*γ*_1_	7.95 × 10^−4^	*H*_o_	14 000.00
$k_{c}^{d}$	1.80	*v*	0.20	*ζ*	1.16

## 4. Results of analyses

### (a) Effect of frequency content of input motion

To investigate the effect of the frequency content of the input motion when P-waves propagate through a level ground deposit, a suite of three analyses were conducted for each seismic event: one models the horizontal component only, one simulates the vertical one and one is *bidirectional* considering the combined effect of both components on liquefaction resistance. All six analyses are first carried out for the 40 m deep FRS deposit.

**Figure 2.**
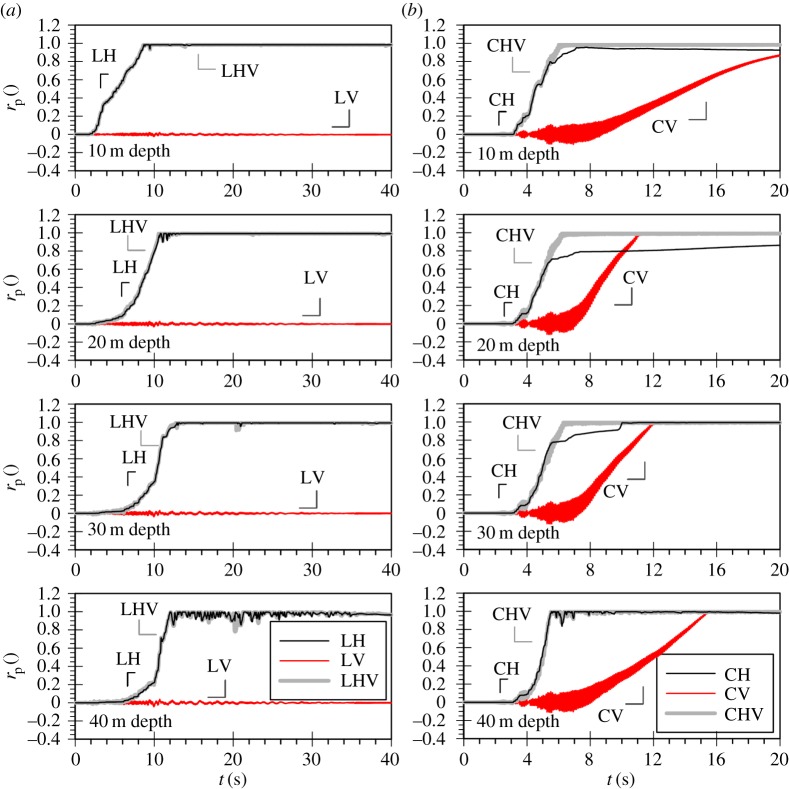
Mean effective stress ratio (*r*_p_) time histories for the (*a*) Lotung and (*b*) Christchurch seismic event during the strong motion.

Figure 2*a,b* shows the mean effective stress ratio, *r*_p_, time histories for the three analyses of the Lotung and of the Christchurch seismic event, respectively. It should be noted that the mean effective stress ratio is defined according to the following equation:

4.1 $$r_{p}=\frac{\Delta p^{\prime }}{p_{o}^{\prime }},$$

where $\Delta p^{\prime }$ is the change in mean effective stress since the start of the analysis and $p_{o}^{\prime }$ is the mean effective stress level after consolidation, prior to the application of the dynamic loading. This is introduced to replace the excess pore water pressure ratio, $r_{u}$, commonly used in liquefaction analyses, defined as the ratio of the change in pore water pressure, Δ*u*, over the vertical effective stress after consolidation, $\sigma_{\mathrm{vo}}^{\prime }$. The latter is not applicable to loading conditions involving vertical motion as in this case there are substantial changes in total stress which is transmitted directly to the water phase, leading to values of Δ*u* many times larger than $\sigma_{\mathrm{vo}}^{\prime }$, even for situations where liquefaction does not take place. The formulation in equation (4.1) considers only effective stress changes and is, therefore, unaffected by such total stress variations. $r_{p}$ values greater than about 0.9 are used in this study to identify the occurrence of liquefaction, with a value of unity corresponding to complete loss of soil's strength, similar to $r_{u}$ (initial liquefaction [38]).

The $r_{p}$ time histories at 10 m intervals shown in figure 2*a* clearly demonstrate that the scaled horizontal component of the Lotung event (denoted as LH) is sufficiently strong to induce significant nonlinearity and liquefaction for the full depth of the 40 m deep FRS deposit. Liquefaction is inferred by the progressive increase of the mean effective stress ratio towards a value of 1. Conversely, the scaled vertical component (denoted as LV), despite its significant amplitude, does not result in any permanent changes to the mean effective stresses, only in high-frequency oscillations due to total stress changes, in agreement with the findings of previous studies. As a result, the response of the deposit in *bidirectional* loading (denoted as LHV) is practically identical to that obtained when only the horizontal component is applied.

Similarly, figure 2*b* shows that the horizontal component of the Christchurch event (denoted as CH) results in liquefaction of the whole depth of the deposit, as shown in figure 2*b*. Contrary to the Lotung case, however, when the Christchurch vertical component is applied on its own (denoted as CV), significant plasticity is observed, resulting in the liquefaction of the whole deposit. High-frequency oscillations are still present in the mean effective stress ratio time histories, but are now accompanied by the development of residual pore water pressures as $r_{p}$ increases gradually towards a value of one. As anticipated, when the two orthogonal components are combined in the analysis (denoted as CHV) liquefaction down to 40 m depth is also predicted. It should be noted that in this case, despite the considerable additional plasticity due to the inclusion of the vertical motion (CV), the loss of strength in the *bidirectional* analysis (CHV) takes place only marginally earlier compared with the horizontal motion analysis (CH). This can be explained considering that the strong part of the motion for both components takes place at relatively similar time instants, with the peak cycle of the vertical excitation marginally preceding that of the horizontal motion.

**Figure 3.**
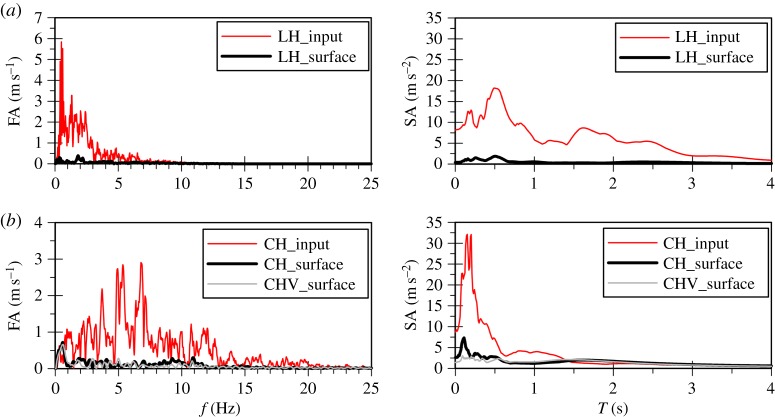
Simulated Fourier and response spectra of (*a*) the Lotung and (*b*) the Christchurch horizontal components from uni-directional (H) and bidirectional (HV) analyses.

**Figure 4.**
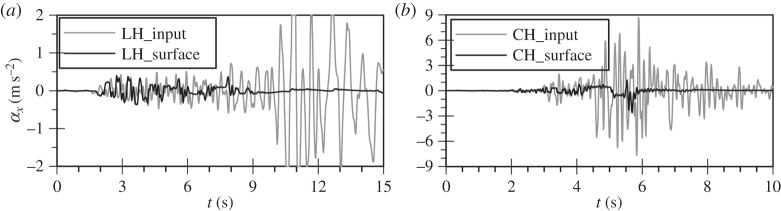
Predicted surface acceleration time histories for the horizontal component analysis of (*a*) the Lotung and (*b*) the Christchurch seismic event.

**Figure 5.**
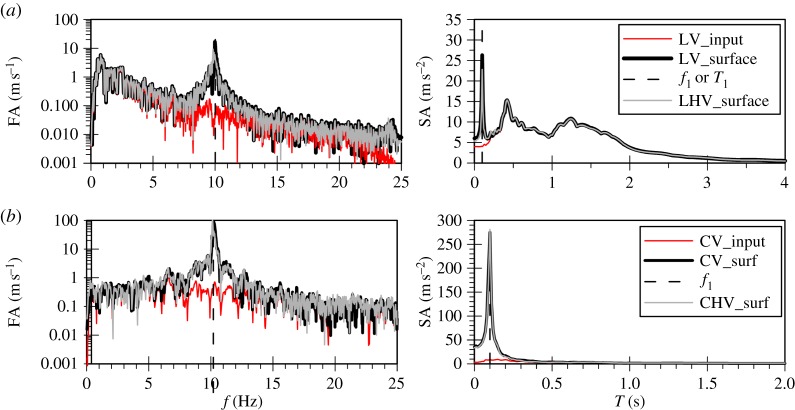
Simulated Fourier and response spectra of (*a*) the Lotung and (*b*) the Christchurch vertical component from uni-directional (V) and bidirectional (HV) analyses.

**Figure 6.**
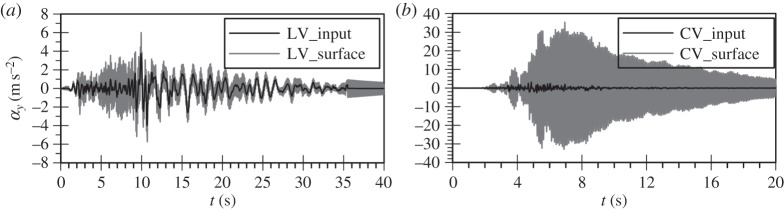
Predicted surface acceleration time histories for the vertical component analysis of (*a*) the Lotung and (*b*) the Christchurch seismic event.

In order to further investigate the physical mechanism underlying the above findings, the surface Fourier and response spectra (spectral acceleration, SA) as well as the surface acceleration time histories for each analysis are compared with those corresponding to the input motions in figures 3 to 6. All response spectra in this study have been calculated for 5% damping of the single degree of freedom system. As expected, the surface response of the horizontal motion of the Lotung and Christchurch events shows de-amplification due to the occurrence of liquefaction. This is clearly evident in the overall reduction in the amplitudes in the computed surface spectra compared with the input ones (figure 3). In fact, in the case of the *bidirectional* analysis for the Christchurch event the additional plasticity due to the vertical motion results in more de-amplification compared with the predictions of the horizontal motion analysis only (figure 3*b*). The reduction in material stiffness due to the nonlinear soil response manifests itself as period elongation, shown in the acceleration time histories. The attenuation of high frequencies is particularly evident for both the Lotung and Christchurch horizontal components after about 5 s of strong motion duration (figure 4).

Contrary to the soil response in the horizontal plane, the stiffness of the deposit in the vertical direction is governed by the bulk modulus of the water which shows no *hysteresis*. Therefore, an amplification at the fundamental frequency of the deposit for P-waves, corresponding to about 10 Hz, is expected to take place. This is in accordance with downhole array field records of vertical motion amplification in liquefiable sites [15,39]. For the Lotung vertical motion the absence of significant input components at about 10 Hz, as shown in figure 5*a*, results in a rather small amplification of the motion towards the surface, with the predicted surface acceleration time history being fairly similar to the input one (figure 6*a*). Conversely, in the case of the Christchurch seismic event, the presence of significant input components close to 10 Hz, where the fundamental frequency of the deposit lies (figure 5*b*), and the consequent amplification of these components by the deposit, due to resonance, leads to the development of high acceleration amplitudes at surface (figure 6*b*). This in turn results in substantial changes in the normal effective stresses. As the model is formulated in generalized stress space and compressible pore fluid is assumed, the above leads to the development of significant deviatoric stresses which result in plastic strains and, therefore, in an increase in pore water pressures.

It should be noted that surface motion amplitudes for the CV analysis remain high even after the end of the strong motion duration at about 10 s, resembling a free vibration (figure 6*b*). This indicates the small levels of damping due to the non-hysteretic behaviour of the water phase. The low values of material damping were confirmed by matching the peak of the transfer function (TF) of the vertical motion analysis to the analytical TF for the steady state solution of a harmonic wave propagating through a visco-elastic soil layer over rigid rock [40]. For this, a damping ratio as small as 0.4% had to be used, proving the original hypothesis. Note that the TF is defined in this study as the ratio between the surface Fourier spectrum and the input one. The above imply that the potential for large surface vertical accelerations due to resonance could be predicted through a simple linear elastic analysis, provided that appropriate values for the damping ratio and the stiffness of the sand deposit are used.

Finally, it is interesting to investigate whether the previous observations could also be justified in terms of cyclic stress ratio (CSR) time histories at various depths in the deposit for the vertical motion analyses. These have been calculated on the basis of the CSR definition for cyclic triaxial tests, as given by the following equation [38]:

4.2 $$\mathrm{CSR}=\frac{q}{2\cdot p_{o}^{\prime }},$$

where *q* is the triaxial deviatoric stress amplitude applied and $p_{o}^{\prime }$ is the mean effective stress level after consolidation. The triaxial deviatoric stress amplitude was obtained from time histories of vertical and horizontal normal effective stresses in the deposit, as:

4.3 $$q=\sigma_{v}^{\prime }-\sigma_{h}^{\prime }.$$

Based on this, the deviatoric stress amplitude was defined as half the range of the triaxial deviatoric stress between two consecutive reversals. When considering the propagation of shear waves in a deposit, it is common practice to calculate CSR from the maximum amplitude of the surface acceleration time history that shows no signs of liquefaction [41]. Nevertheless, in the case of P-wave propagation, the assumption regarding the hydraulic phase has significant implications on the natural frequencies of the system, because the drained response is controlled by the soil compressibility which is usually significantly different from that of the fluid. Therefore, the CSR was calculated from the coupled hydro-mechanical analyses. Consequently, any development of excess pore water pressures will result in a reduction in the calculated CSR which would not have been seen had the analysis been drained.

**Figure 7.**
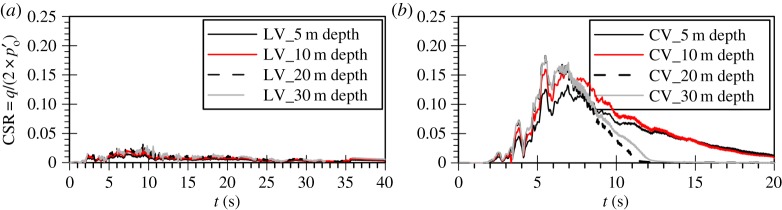
Time histories of predicted cyclic stress ratio for the vertical component of (*a*) the Lotung and (*b*) the Christchurch seismic event.

Figure 7*a* shows the cyclic stress ratio time histories for the Lotung vertical component analysis. As expected, the CSR values are very small for the whole depth of the deposit, justifying the linear elastic response previously seen. Contrary to that, the cyclic stress ratio amplitudes that develop in the case of the Christchurch vertical component analysis, as shown in figure 7*b*, are significantly higher, taking values up to almost 0.2. Based on the cyclic strength curves for FRS as obtained from direct simple shear tests by Sriskandakumar [36], as well as the numerical cyclic strength curves as established by Klokidi [34], these values of CSR are sufficiently large to induce plastic response and liquefaction. It is also worth noting that the distribution of CSR amplitudes with depth exhibits an increasing trend with deeper levels. This could justify the observed pattern in the progression of the liquefaction front in the Christchurch vertical motion analysis, which does not initiate from the top of the deposit (figure 2).

**Figure 8.**
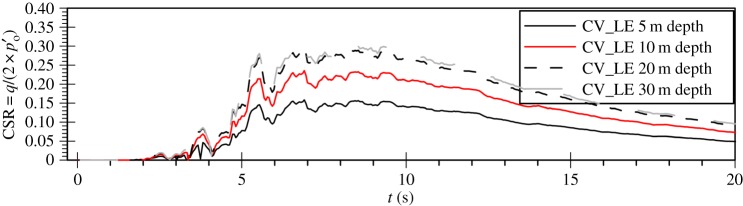
Time-histories of predicted cyclic stress ratio for the vertical component of the Christchurch seismic event in linear elastic FE analysis.

To ensure that the observed trend is not influenced by the constitutive model, a linear elastic coupled hydro-mechanical FE analysis with ICFEP was carried out for the Christchurch vertical component using the same mesh and boundary conditions. Because for the vertical motion the response is governed by the bulk stiffness of the water and no nonlinearity is observed, the response in terms of amplification can readily be obtained from a simple linear elastic analysis. A constitutive model which allows for a spatial variation of the shear modulus, *G*, and the bulk modulus, *K*, was used to simulate the initial stiffness profile in the 40 m deep FRS deposit [24]. For simplicity, due to the large amplification which implies practically no *hysteresis*, no Rayleigh damping was used in the analysis. The results are shown in figure 8, confirming the previously seen trend of increasing CSR with increasing depth in the deposit.

### (b) Effect of depth of sand deposit

To further test the hypothesis of resonance in the case of the vertical seismic motion and sand liquefaction, the analysis with the Lotung vertical component was repeated for a deeper deposit of 166 m depth. This was done to shift the natural frequency of the deposit from a value of 10.3 Hz to 2.46 Hz and hence, to a region where significant components in the Lotung motion exist (figure 1*b*).

**Figure 9.**
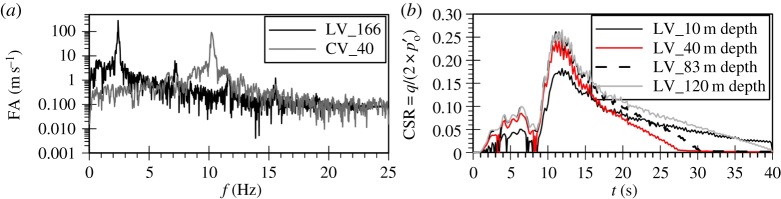
(*a*) Simulated surface Fourier spectrum and (*b*) time-histories of predicted cyclic stress ratio for the vertical component of the Lotung seismic event—166 m deep FRS deposit (LV_166).

As expected, resonance now takes place and the input components at about 2.5 Hz are amplified significantly towards the surface (figure 9*a*), leading to the development of significant cyclic stress ratios for the whole depth of the deposit, as shown in figure 9*b*.

**Figure 10.**
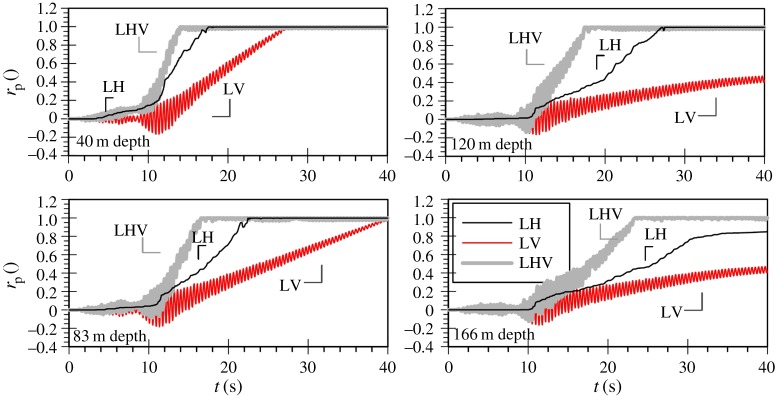
Mean effective stress ratio (*r*_p_) time histories for the Lotung seismic event during the strong motion—166 m deep FRS deposit.

Figure 10 shows the mean effective stress ratio time histories at various depths in the 166 m deep FRS deposit. Similar to the patterns observed for the 40 m deep deposit subjected to the Christchurch vertical motion, plasticity develops throughout the entire depth and liquefaction occurs down to about 110 m depth.

Two more analyses with the 166 m deep FRS deposit were carried out and are presented in figure 10: one with the Lotung horizontal component (LH) and one *bidirectional* (LHV). Again the horizontal motion induces nonlinearity, with the occurrence of liquefaction being observed down to about 150 m depth. It is interesting to note that in this case, the additional plasticity due to the vertical motion in the 166 m deep deposit results in earlier liquefaction triggering when the two components are combined in the analysis (LHV), when compared with the predictions of the horizontal motion only (LH). The maximum liquefaction zone in LHV is now also increased to include the full depth of the deposit. These effects were negligible in the case of the shallow deposit.

### (c) Effect of hydraulic regime

All analyses so far have been conducted with a constant permeability value. Theoretical and experimental evidence, however, suggests that the permeability of a saturated sand deposit subjected to earthquake loading changes and this is believed to be attributed to variations in the effective porosity of the soil mass and in the tortuosity of the flow paths as a result of the formation of transient cracks [42,43]. In particular, it has been shown through the study of observations from centrifuge tests that, due to the agitation effect, permeability under dynamic excitation increases rapidly close to the state of liquefaction, when $r_{u}$ or $r_{p}$ approach a value of one [32,43–46]. The coefficient of permeability has been shown to influence substantially the rate of build-up and magnitude of excess pore water pressures, as well as the amount of volumetric deformation during shaking [46]. Therefore, the conventional assumption of undrained response of saturated sand deposits during earthquake loading is questionable, with co-seismic settlements shown to be significant due to upward flow of water and water discharge from the soil mass as permeability increases [32,43,46–48].

Previous researchers who have investigated the effects of increased permeability during liquefaction have either used constant increased hydraulic conductivity values [42,48,49] or variations of permeability with time [50–52]. Su *et al*. [46] back-calculated the permeability at liquefaction in a dynamic centrifuge test based on the observed surface settlements and the variation of excess pore water pressures in the model using the law of conservation of mass. The analytical results showed that an increased ‘in-flight’ permeability of six times the static value would be required to give the observed surface settlement rate. Numerical analyses using a constant increased permeability verified the conclusions. Taborda [32] back-calculated the permeability at liquefaction for VELACS Model 1 [53] in a similar manner and obtained a maximum permeability at liquefaction seven times larger than the initial one. Subsequent FE analyses using a variable permeability model in which the permeability is a function of the excess pore water pressure ratio, $r_{u}$, confirmed the improved predictions compared with analyses using a constant static value [32]. Shahir *et al*. [54] also linked the variation of permeability to the excess pore water pressure ratio in FE analyses reproducing liquefaction in centrifuge tests. A similar expression was also used by Chaloulos *et al*. [55] to simulate lateral spreading. From observations from experiments involving a range of centrifuge and shaking table tests, Shahir *et al*. [47] concluded that the in-flight permeability during liquefaction is 10–14 times larger than the static one.

**Figure 11.**
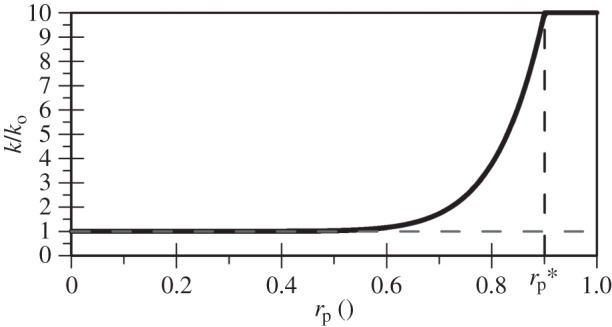
Modelled variation of permeability with mean effective stress ratio.

To investigate the effect of the hydraulic regime on liquefaction triggering as well as post-liquefaction settlements due to the inclusion of the vertical component in the simulations, a set of three extra analyses with variable permeability were also carried out for the shorter FRS deposit and the Christchurch motion: one with the horizontal component only (denoted as CH_Vk), one with the vertical component only (denoted as CV_Vk) and one *bidirectional* analysis (denoted as CHV_Vk). To reproduce numerically this feature of dynamic soil response, a nonlinear variable permeability model is used, in which the permeability is a power function of the mean effective stress ratio, $r_{p}$, according to the following equation:

4.4 $$\frac{k}{k_{o}}=1+\left(\frac{k_{\max}}{k_{o}}-1\right)\cdot {\left(\frac{r_{p}}{r_{p}^{*}}\right)}^{n_{k}},$$

where $k_{o}$ is the initial static permeability as measured in conventional laboratory testing, $k_{\max}$ is the maximum permeability at the time of liquefaction $\left(r_{p}\approx 1\right)$ and $n_{k}$ is a model parameter which governs the nonlinearity of the effect of varying $r_{p}$. For a ratio of $\;k_{\max}$ over $k_{o}$ equal to 10 and $n_{k}$ and $r_{p}^{*}$ equal to 10 and 0.9, respectively, the distribution depicted in figure 11 is obtained. It can be shown that the latter parameters ensure a variation of *k* between $r_{p}$ values of 0.6 and 0.9, with *k* obtaining a maximum value of 4.2 × 10^−3 ^m s^−1^. The above expression is similar to that used by Taborda [32], with the only difference residing in the incorporation of $r_{p}$ instead of $r_{u}$ originally used, to avoid concerns over total stress changes, as previously explained.

It should be mentioned that assuming a constant permeability equal to the maximum one (4.2 × 10^−3 ^m s^−1^) and based on Zienkiewicz's *et al*. [56] analytical solution of Biot's equations for a laterally infinite soil medium [57,58], the response of the 40 m deep FRS deposit subjected to the Christchurch horizontal component corresponds to undrained behaviour. Conversely, when subjected to the Christchurch vertical component, the response lies within Zone II, where the ‘u-p’ formulation is valid [56], but some drainage is expected to take place.

**Figure 12.**
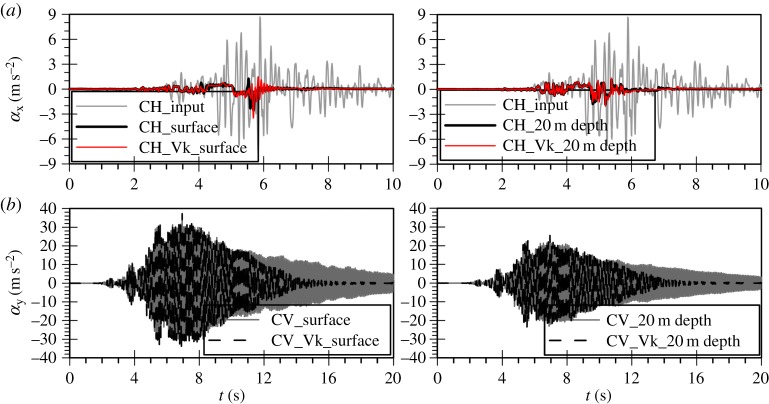
Acceleration time histories at 0 and 20 m depth from uni-directional analyses of (*a*) the horizontal and (*b*) the vertical component of the Christchurch seismic event—constant (CH, CV) versus variable permeability analysis (CH_Vk, CV_Vk).

In figure 12*a*, the simulated horizontal acceleration time histories at 0 and 20 m depth are compared with those from the corresponding constant permeability analysis (CH). No substantial difference can be seen with amplitude decay and liquefaction taking place at similar time instants.

Similarly, figure 12*b* shows the corresponding comparison for the vertical acceleration time histories, indicating that acceleration response is similar up to about 10 s, but then reduces more rapidly in the case of the variable permeability analysis.

**Figure 13.**
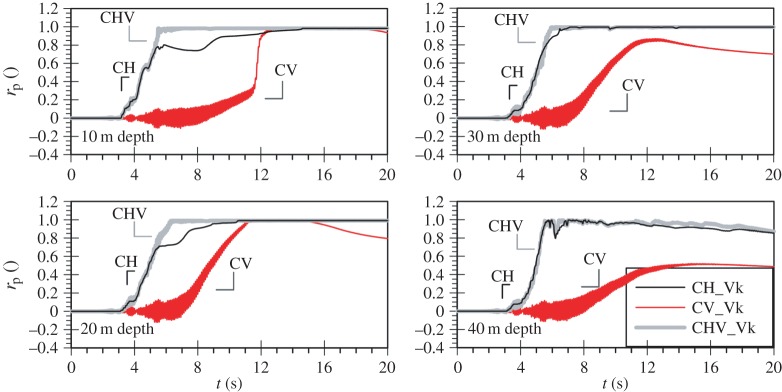
Mean effective stress ratio (*r*_p_) time histories during the strong motion for variable permeability analyses of the Christchurch seismic event.

The above findings can be understood by looking at the mean effective stress ratio time histories for the variable permeability analyses, shown in figure 13. The predictions of the vertical motion analysis show that $r_{p}$ starts attaining values larger than 0.6 towards the end of the strong motion at approximately 10 s for most depths within the deposit. This implies that the permeability retains its static value up to this point, increasing rapidly from then onwards and up to $r_{p}$ values of 0.9. Owing to this increase, the permeability coefficient becomes larger than 1.0 × 10^−3 ^m s^−1^. Han [59] quantified the values of viscous damping by comparing the results of linear elastic fully coupled FE analyses simulating the vertical motion with those of one-dimensional total stress analytical solutions that matched the amplification predictions of the FE simulations. It was shown that, for values of permeability higher than about 1.0 × 10^−3 ^m s^−1^, viscous damping can be quite significant, reaching values up to approximately 10%. This is almost two orders of magnitude larger than the hysteretic damping in the vertical direction discussed earlier for the constant permeability analyses, leading to the de-amplification of the vertical acceleration in the variable permeability analysis. The linear elastic FE analyses in the study of Han [59] also showed such an effect of viscous damping on the vertical amplification.

**Figure 14.**
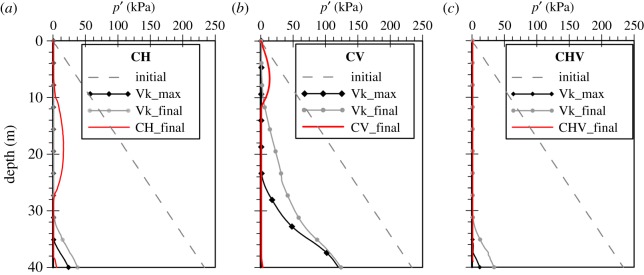
Mean effective stress profiles registered during the strong motion for (*a*) the horizontal, (*b*) the vertical and (*c*) bidirectional analysis of the Christchurch seismic event for variable (Vk) and constant permeability analyses.

From figure 13, it is also evident that variable permeability and the resulting drainage mostly affects the deeper parts of the deposit, agreeing with the findings of Su *et al*. [46] who compared the results of numerical analyses of various constant permeability values. This is also shown in figure 14, where the mean effective stress profiles, corresponding to the maximum depth of liquefaction (Vk_max) and to the end of the strong motion (Vk_final), are shown separately for the three variable permeability analyses. The profiles at the end of the strong motion for the corresponding constant permeability analyses have also been superimposed on the graphs. These also represent the maximum depth of liquefaction, as hardly any drainage took place in those analyses. From these, it can be seen that, due to the higher flow of water upwards and drainage during the strong motion, the maximum liquefaction depth in the CV_Vk analysis appears to be reduced compared with the original constant permeability analysis: 25 m in the former compared with 40 m, i.e. the entire depth, in the latter. The final liquefaction depth at 20 s also appears to be reduced in CV_Vk analysis down to approximately 10 m. Similar are the predictions for CH_Vk and CHV_Vk with reduced final liquefaction depths at 20 s down to about 34 m depth. Despite the additional drainage due to the increase in the hydraulic conductivity, the maximum liquefaction depth in both analyses (CH_Vk, CHV_Vk) is 40 m, similar to the predictions of the corresponding constant permeability analyses (figure 14). It is to be noted that, in accordance with the predictions of Zienkiewicz *et al*. [56] theory presented earlier, for the considered cases drainage is expected to affect the higher frequency vertical motion analysis' results more compared with the horizontal one.

Owing to the increase in permeability and the higher upward water flux, particularly at shallower depths in the deposit, liquefaction in the *bidirectional* analysis takes place slightly earlier compared with the horizontal motion analysis (figure 13). At deeper depths, however, where the flow of water and the accumulation of excess pore water pressures due to vertical motion are not as substantial, the predictions of the horizontal motion and the *bidirectional* analyses are fairly similar.

**Figure 15.**
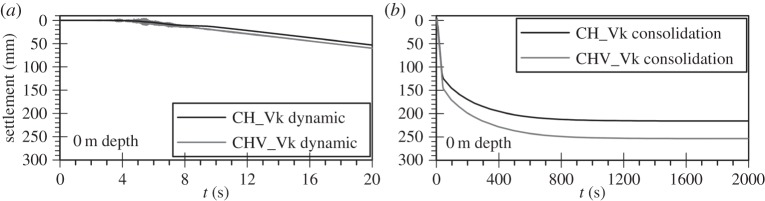
Predicted (*a*) co-seismic and (*b*) post-consolidation surface settlements for the uni-directional (horizontal component CH) and bidirectional analysis (CHV) of the Christchurch seismic event for variable permeability (Vk) analyses.

The final aspect of the variable permeability analyses investigated is the prediction of the co-seismic and post-liquefaction surface settlements considering the horizontal motion and the *bidirectional* analyses (figure 15). It can be seen that both analyses predict a similar amount of settlement during shaking, with co-seismic values being up to about 60 mm. It is, however, interesting to note that, despite the predictions of the maximum depth of liquefaction and liquefaction triggering being similar between the two analyses, post-liquefaction permanent settlements in the *bidirectional* analysis appear to be increased by approximately 25% compared with the horizontal motion only. This is considered to be a result of the additional plasticity induced by the inclusion of the vertical motion in the simulation.

## 5. Strong motion stations in Christchurch, New Zealand

**Figure 16.**
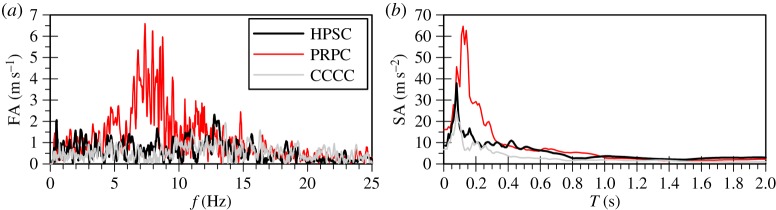
Measured surface (*a*) Fourier spectra and (*b*) response spectra of the vertical component of 22 February 2011 Christchurch seismic event for strong motion stations HPSC, PRPC and CCCC.

To verify the hypothesis of resonance in the case of the vertical seismic motion, the surface vertical acceleration time histories for the strong motion stations of Hulverstone Drive Pumping (HPSC), Pages Road Pumping (PRPC) and Christchurch Cathedral College (CCCC) in Christchurch, New Zealand, where significantly high vertical accelerations were registered during the 22 February 2011 seismic event [7], were analysed [19]. Figure 16 shows the surface Fourier and response spectra, with the peak response corresponding to frequencies of 12.75, 7.4 and 13.6 Hz or periods of 0.078, 0.135 and 0.074 s for stations HPSC, PRPC and CCCC, respectively. These correspond to a frequency range similar to the fundamental frequency for P-waves of the 40 m deep FRS deposit used in the current study, where significant components in the input vertical motion exist.

The above peak responses are not surprising if one considers the stratigraphy in Christchurch, with alluvial and marine sandy deposits approximately 10–30 m thick, overlying the stiffer Riccarton Gravel horizon [61]. At the locations of HPSC, PRPC and CCCC, in particular, the depth to gravel corresponds to about 35, 30 and 22 m, respectively, with the GWTL close to the ground surface [62], meaning that natural periods as those shown above would be expected when considering the vertical seismic motion. As such, resonance could be a realistic scenario justifying the large vertical accelerations recorded in Christchurch. Lee *et al*. [12] also quote the peak surface response of the vertical motion of the Christchurch event to correspond to a period of about 0.08 s, agreeing with the above conclusions.

Additionally, measurements of P-wave velocity below the ground water table in a number of sites close to the above SMS were obtained from the Canterbury Geotechnical Database [63]. These were found to correspond to those of a fully saturated deposit, implying that the scenario of partial saturation below the water table does not seem to hold true in this case.

## 6. Conclusion

This study focuses on the implications of vertical ground motion, and in particular, compressional waves, as well as *bidirectional* earthquake loading on the liquefaction response of fully saturated sand deposits. The frequency content of the input excitation, the depth of the deposit and the variable permeability during liquefaction have been investigated with the results contradicting some of the findings of previous studies. These are summarized below.

When the vertical ground motion is rich in frequencies in the range where the fundamental frequency of the deposit for P-waves lies, resonance can occur leading to the development of significant deviatoric stresses which in turn can induce plasticity and, if sufficiently strong, may lead to soil liquefaction. Peak ground acceleration of the input vertical motion appears to be not an appropriate parameter for damage evaluation.

The commonly adopted assumption of linear elastic behaviour when compressional waves propagate vertically upwards in a saturated sand deposit is not valid in such cases of resonance.

When the two components (i.e. vertical and horizontal) are combined in the analysis, increased plasticity can be engaged. This can increase the maximum depth of liquefaction and can lead to liquefaction triggering taking place earlier in the strong motion duration.

Even in the case where liquefaction in the *bidirectional* analysis occurred marginally earlier compared with uni-directional horizontal motion analysis and the maximum depth of liquefaction was unaltered, the inclusion of the vertical component led to larger post-liquefaction surface settlements.
